# Effect of resistance training on body composition and physical function in older females with sarcopenic obesity—a systematic review and meta-analysis of randomized controlled trials

**DOI:** 10.3389/fnagi.2025.1495218

**Published:** 2025-04-30

**Authors:** Changsheng Guo, Ting Dai, Haoran Zhang, Meng Luo, Jing Gao, Xiaodong Feng

**Affiliations:** ^1^Heilongjiang University of Chinese Medicine, Harbin, China; ^2^Rehabilitation Center, The First Affiliated Hospital of Henan University of Chinese Medicine, Zhengzhou, China; ^3^Department of Rehabilitation Medicine, Henan University of Chinese Medicine, Zhengzhou, China

**Keywords:** resistance training, sarcopenic obesity, older females, body composition, physical function

## Abstract

**Objectives:**

A systematic review and meta-analysis was conducted to validate the effects of resistance training (RT) on body composition and physical function in older females with sarcopenic obesity (SO).

**Design:**

Systematic review and meta-analysis.

**Setting and participants:**

Older females (≥60 years).

**Methods:**

Four electronic databases—PubMed, Web of Science, Embase, and the Cochrane Library—were comprehensively searched through June 2024. Randomized controlled trials (RCTs) comparing RT with non-exercise interventions or health education were included. Outcomes measured included key indicators such as body composition and physical function. The quality of the included studies was evaluated using the Physiotherapy Evidence Database (PEDRO) score, and the risk of bias was assessed utilizing the Cochrane Risk of Bias 2.0 Tool (RoB 2). Ultimately, a meta-analysis was conducted using RevMan 5.4.

**Results:**

Results of our meta-analysis revealed that RT partially ameliorated body composition in patients, significantly reducing body fat percentage (BF%; WMD = −2.83, 95% CI: −4.55 to −1.12, *p* = 0.001). However, through comparative analysis of the control groups, we revealed that it did not significantly influence other indices such as body mass index (BMI; WMD = −0.42, 95% CI: −1.92 to 1.08, *p* = 0.58), total skeletal muscle mass (TSM; WMD = −0.62, 95% CI: −2.38 to 1.15, *p* = 0.49), or bone mineral density (BMD; WMD = 0.01, 95% CI: −0.03 to 0.05, *p* = 0.68). Notably, RT demonstrated substantial efficacy in enhancing physical function, as evidenced by improvements in the 10-meter walk test (10WMT; WMD = 0.22 s, 95% CI: 0.04 to 0.39, *p* = 0.01), Timed Up and Go test (TUG; WMD = −2.23 s, 95% CI: −2.96 to −1.49, *p* = 0.00001), and Timed Chair Rise test (TCR; WMD = 5.20 repetitions, 95% CI: 3.98 to 6.43, *p* = 0.00001).

**Conclusion:**

This meta-analysis indicates that RT exerts a significant positive influence on the physical function of older females with SO. Despite these benefits, the impact on body composition parameters, such as BF%, appears to be limited. These findings underscore the need for further investigation into the mechanisms by which RT affects body composition in this patient population.

**Systematic review registration:**

INPLASY202430061 https://inplasy.com/inplasy-2024-3-0061/.

## 1 Introduction

Obesity, a major public health challenge of the 21st century, transcends age boundaries and now manifests as a global pandemic affecting the entire human population (Gavras and Batsis, 2024). The older population may experience an elevated risk of obesity due to declines in basal metabolic rate (BMR), alterations in body composition, and reduced physical mobility (Javed et al., 2020). Sarcopenic obesity (SO) is aptly defined as a novel phenotype arising from the concurrent aging of the population and the obesity epidemic (Lee, 2024), characterized primarily by obesity accompanied by a decline in muscle mass and function (Villareal et al., 2004). Skeletal muscle loss is primarily driven by reduced physical activity levels and insufficient protein intake. These metabolic deficits interact with aberrant adipose tissue hyperplasia to establish a bidirectional vicious cycle, collectively exacerbating the progression of obesity (Villareal et al., 2004). Both sarcopenia and obesity independently correlate with physical disabilities in the older people; However, SO results in greater physical limitations than either sarcopenia or obesity alone (Dominguez and Barbagallo, 2007; Stenholm et al., 2008). Studies have confirmed that older females are approximately three times more likely to suffer from SO than older men (Jang and Kim, 2023). This disparity may be attributed to the natural decline in muscle mass, strength, and function that accompanies aging. Additionally, the significant drop in estrogen levels post-menopause in older females increases the hypothalamic release of gonadotropin-releasing hormone (GnRH), which subsequently stimulates the anterior pituitary to produce follicle-stimulating hormone (FSH) (Barbagallo et al., 2024). FSH then binds to the Gi protein *α*-subunit (Gαi), an inhibitory coupling protein of the FSH receptor (FSHR) expressed in visceral adipose tissue (VAT), promoting lipid biosynthesis (Liu et al., 2015). This process makes SO more prevalent in older females.

In recent years, SO has attracted extensive attention from researchers due to multiple factors: its explosive growth against the dual backdrop of population aging and metabolic diseases, the mechanism of muscle-fat interaction imbalance that has overturned traditional understanding, and the persistent clinical challenge of balancing muscle gain and weight reduction in patients (Zhang et al., 2024). Nonetheless, its intricate pathogenesis has left us without approved medications for treatment, presenting notable social and economic challenges (Assyov et al., 2024). Non-invasive treatments are recognized for their wide applicability, relative safety, and cost-effectiveness; among these, exercise training has gradually gained public attention as a crucial method (Arora et al., 2024).

Resistance training (RT), a crucial element of exercise regimens, is distinguished by its high intensity, brief duration, and exhaustive nature, thereby exhibiting significant efficacy in enhancing muscle strength, quality, and overall physical fitness in the older populations (Lee et al., 2024; Winters-Stone et al., 2024; Sinclair et al., 2024). It has been extensively utilized in the management of sarcopenia and obesity. Nonetheless, given that SO involves more intricate physiological and pathological mechanisms compared to simple sarcopenia or obesity, it remains uncertain whether RT can serve as a primary non-pharmacological treatment with definitive efficacy. A recent systematic review by Debes et al. (2024) and Ghiotto et al. (2022) indicated that RT can significantly improve various outcomes in older females with SO, including body composition, muscle strength, and functional ability. However, related RCT results present contradictions, suggesting that while RT is effective for certain body composition parameters, such as total fat-free mass (TFFM), it is ineffective for indicators like body fat percentage (BF%) and body mass index (BMI) (Gadelha et al., 2016). Given the inconsistency of experimental results, the therapeutic potential of RT remains indeterminate, underscoring the need for comprehensive comparative studies to elucidate its unique benefits.

Given these considerations, it is imperative to update the systematic review and meta-analysis to evaluate the comparative efficacy of RT in managing SO. Thus, this study seeks to undertake a systematic review and meta-analysis of recent randomized controlled trials (RCTs) to assess the therapeutic effects of RT on SO. The investigation will primarily focus on examining the influence of this intervention on body composition and physical function in older females with SO.

## 2 Methods

### 2.1 Registration

The systematic review and meta-analysis adhered to the Preferred Reporting Items for Systematic Reviews and Meta-Analyses (PRISMA) guidelines (Page et al., 2021). This study had been previously reviewed and registered on the INPLSASY platform under registration number INPLASY202430061.

### 2.2 Search strategy

Two independent researchers (CS G and T D) searched in the following four databases (PubMed, Web of Science, EMBASE, and Cochrane Library), spanning from the inception of these databases until June 2024. We conducted the search on Boolean logic using the following terms: (“sarcopenia” OR “sarcopenic”) AND (“obese” OR “obesity” OR “overweight”) AND (“Women” OR “Female”) AND (“Resistance Training” OR Resistance Exercise) AND (“randomized controlled trial” OR “RCT”). And the complete searching strategies of all databases are given in Supplementary Table 1.

### 2.3 Study inclusion and exclusion criteria

The inclusion and exclusion criteria were developed following the PICOS principles (Participants, Intervention, Comparison, Outcome, and Study). (1) Population and disease (P): The subjects were females with sarcopenic obesity; Age: ≥60 years old; Patients with severe other complications such as cancer, multiple sclerosis, strokes, cognitive impairment were excluded. (2) Intervention (I): The intervention was resistance exercise, including but not limited to muscle training, progressive strength and/or resistance training, weight training and/or elastic band training; Two or more comprehensive interventions other than resistance training were excluded. (3) Comparison (C): the control group was non-exercise treatment or telephone follow-up. (4) Outcome (O): Analysis of body composition and/or physical function were included; The outcome indicators did not match were excluded. (5) Study (S): Randomized controlled studies were included; Other types of experiments, case reports and systematic reviews were excluded. Other: Only English-language studies published before 31 June 2024, were included; publications such as reviews, pathology reports, conference abstracts, and letters were excluded.

### 2.4 Data extraction

First, EndnoteX9 software was employed to manage the search records and eliminate duplicates. Two independent researchers (HR Z and M L) then conducted an initial screening of the identified literature by reviewing abstracts and titles. The same researchers conducted a full-text review of the remaining studies, resolving any disagreements through consultation with a third-party mediator. The extracted data included publication details (authors, year of publication, and country), subject characteristics (number of participants, age, and BMI), definitions of SO, assessment tool of body composition, intervention programs (type of intervention, control group intervention, Frequency, intensity, duration, supervision status, and adherence), and outcome indicators. Details are provided in Table 1.

**Table 1:** Characteristics of included studies.

Author and year	Country	N (Exp/Con)	Age	BMI (Exp/Con)	Definition of sarcopenic obesity		Assessment tool of body composition	Intervention measures	Control measures	Frequency	Intensity	Time	Supervision	Adherence (%)	Clinical measure (s)
					Obesity	Sarcopenia									
Liao et al. (2017)	China	25/21	(60–80 years)	27.32 ± 3.33/28.19 ± 3.27	BF% > 30	SMI (TSM/Ht2) < 7.15 kg/m2	BIA	Elastic resistance exercise	no exercise intervention	3 day/w, 12 weeks	RPE (6–20): 10–13	35–40 min/day，3 sets of 10，15 or 20reps	Yes	97.6%	①⑤⑥⑦
Liao et al. (2018)	China	33/23	(60–80 years)	27.27 ± 3.72/29.16 ± 3.62	BMI > 30 kg/m^2^	SMI (TSM/BW) < 27.6%	BIA	Elastic band exercise	no exercise intervention	3 day/w, 12 weeks	RPE (6–20): 10–13	40 min/day, 3 sets of 10 or 15 reps	Yes	97.6%	①②⑤⑥⑦
Banitalebi et al. (2020)	Iran	32/31	(65-80 years)	33.72 ± 3.15/32.53 ± 2.01	BF % > 32%, BMI > 30kg/m^2^	SMI < 28 or ≤7.76 kg/m^2^	DXA	Elastic band resistance exercise	telephone con-tacts or face-to-face interviews	3 day/w, 12 weeks	RPE (6–20): 10–12	60 min/day, 1–2 sets of 12 reps	Yes	85	①③④
Vasconcelos et al. (2016)	Brazil	16/15	(60–80 years)	32 ± 2.3/33 ± 2.9	BMI≥30kg/m^2^	HG ≤ 21 kg	NA	Resistance exercise program	Monitored by telephone calls once a week	2 day/w, 10 weeks	50–75% (1RM)	60 min/day, 2–3 sets of 8–12 reps	Yes	85%	⑥
Cunha et al. (2018)	Brazil	23/22	(≥60 years)	26.7 ± 4.8/26.7 ± 4.6	NA	NA	DXA	Resistance training	no exercise intervention	3 day/w, 12 weeks	NA	50 min/day, 3 sets of 10–15 reps	Yes	85%	①④
Huang et al. (2017)	China	18/17	(≥60 years)	27.31 ± 3.74/28.96 ± 3.49	BMI≥30kg/m^2^	SMI (TSM/BW) < 27.6%	BIA	Elastic band resistance exercise	received only a 40-min lesson about the exercise concept	3 day/w, 12 weeks	RPE (6–20): 10–13	40 min/day, 3 sets of 10 reps	Yes	NA	①②③④
Lee et al. (2021)	China	15/12	(60–90 years)	26.95 ± 3.31/28.93 ± 3.55	BF% > 35	DXA < 5.67% kg/m^2^ and a grip strength of <20 kg or gait speed (GS) of <0.8 m/s	DXA	Elastic band resistance exercise	no exercise intervention	3 day/w, 12 weeks	RPE (6–20): 10–13	40 min/day，3 sets of 10 reps	Yes	NA	①②④⑤⑥⑦

BIA, bioimpedance analysis; DXA, dual X-ray absorptiometry; ① BF%, Body fat percentage; ② TSM, Total skeletal muscle mass; ③ BMI, Body mass index; ④ BMD, Bone mass density; ⑤ TUG, Timed up and go; ⑥ 10MWT, 10-min walk test; ⑦ TCR, Timed chair rise.

### 2.5 Risk of bias assessment

**Figure 1:**
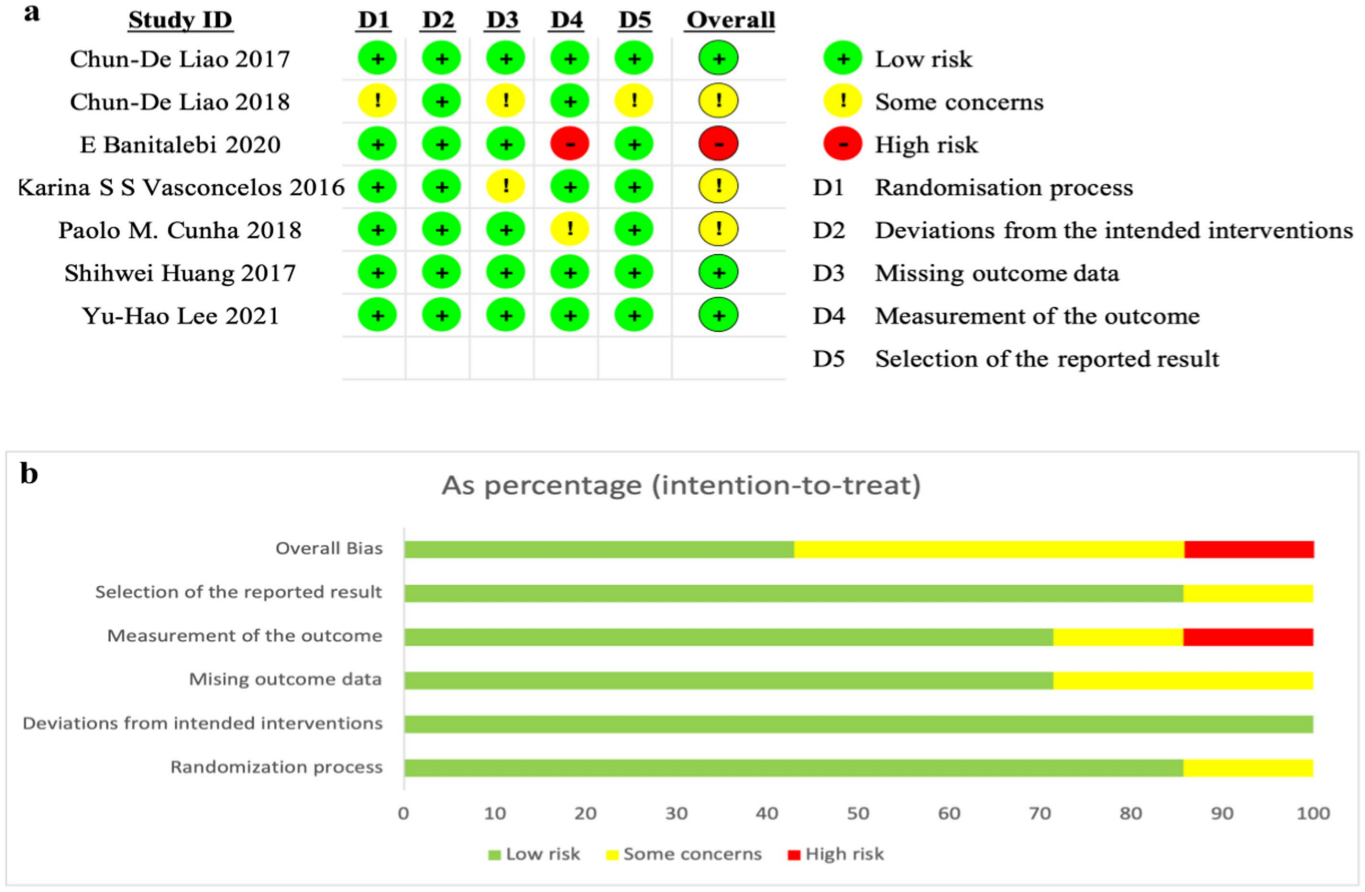
Risk of bias. **(a)** Risk of bias assessment for included studies; **(b)** judgments on each domain as a percentage.

The methodological quality was assessed using the Cochrane Risk of RoB 2 (Figure 1; Sterne et al., 2019). Two investigators independently evaluated the risk of bias in the included studies, which includes five domains. Each domain in all trials was assigned a study-level score indicating the level of bias risk: low, high, or unclear. Discrepancies in assessments were resolved through reviewer discussions, with specific evidence from the studies referenced to clarify evaluations. When consensus could not be achieved through deliberation, a third independent reviewer was consulted to adjudicate. Funnel plots were not generated due to the insufficient number of studies.

### 2.6 Methodological quality of the studies

The methodological quality of the studies was performed with the Physiotherapy Evidence Database (PEDro) scale. We used the Grades of Recommendation, Assessment, Development, and Evaluation (GRADE) criteria to rate the quality of evidence for the outcomes (Cashin and McAuley, 2020). The methodological quality of all included studies is shown in Table 2.

**Table 2:** Methodological quality indicated by PEDro criteria of the included studies.

Study	(1) Eligibility criteria were specified	(2) Random allocation	(3) Concealed allocation	(4) Groups similar at baseline	(5) Participant blinding	(6) Therapist blinding	(7) Assessor blinding	(8) Intention-to-treat analysis (>85%)	(9) Intention-to-treat analysis	(10) Between group difference reported	(11) Point estimate and variability reported	Score
Liao et al. (2017)	Y	Y	N	Y	N	N	Y	Y	Y	Y	Y	7
Liao et al. (2018)	Y	Y	N	Y	N	N	N	Y	Y	Y	Y	6
Banitalebi et al. (2020)	Y	Y	Y	Y	N	N	N	Y	Y	Y	Y	7
Vasconcelos et al. (2016)	Y	Y	N	Y	N	N	N	Y	Y	Y	Y	6
Cunha et al. (2018)	Y	Y	N	Y	N	N	N	Y	Y	Y	Y	6
Huang et al. (2017)	Y	Y	Y	Y	N	N	Y	Y	Y	Y	Y	8
Lee et al. (2021)	Y	Y	Y	Y	N	N	Y	Y	Y	Y	Y	8

Y, one point; N, score 0. A total PEDro score is achieved by adding the ratings of (2) to (11) for a combined total score between 0 and 10.

### 2.7 Data analysis

RevMan 5.4 software was used to assess the risk of bias in the studies and to perform a meta-analysis of the extracted data. The outcome measures in this study are continuous variables, so the standardized mean difference (SMD) or mean difference (MD) is used as the effect size, with a 95% confidence interval (CI) calculated. The Q test combined with the *I*^2^ test was used to assess the statistical heterogeneity between studies. *I*^2^ > 50% and *p* < 0.1 indicate statistical heterogeneity between studies, and a random-effects model was used for analysis. *I*^2^ ≤ 50% and *p* ≥ 0.1 indicate no statistical heterogeneity between studies, and a fixed-effects model was used for analysis. Additionally, we performed a sensitivity analysis for moderately heterogeneous measures, and if the heterogeneity was too large, a descriptive analysis was conducted. Sensitivity analysis was performed by eliminating individual studies one by one to evaluate the robustness of the results.

## 3 Results

### 3.1 Study search results

**Figure 2:**
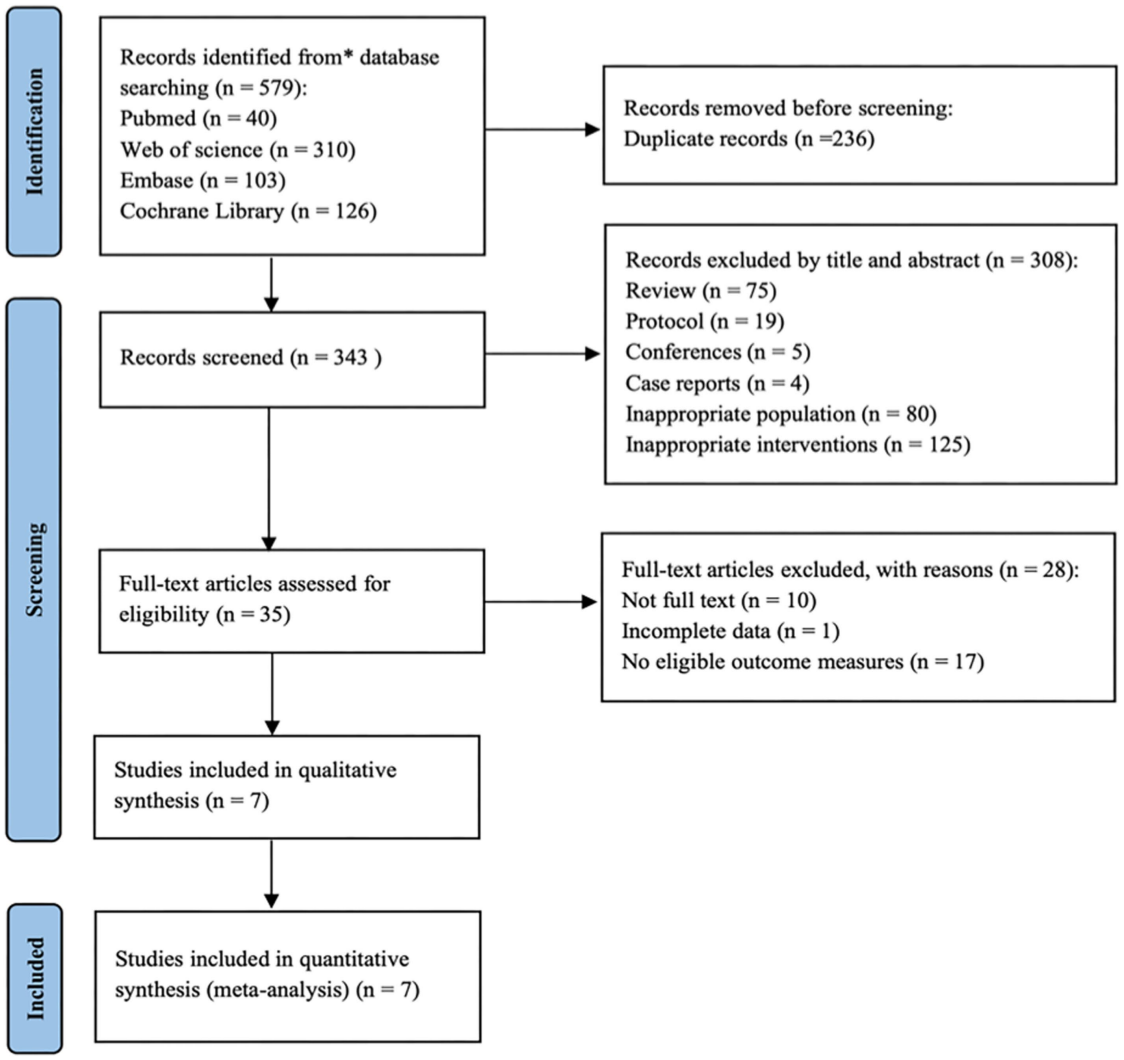
The flowchart of study selection.

A total of 579 studies were identified from four databases based on the pre-established search strategy. After removing 236 duplicate records, two researchers (HR Z and J G) excluded 308 clearly irrelevant records based on titles and abstracts. Then, the eligibility of 35 studies was further evaluated by reviewing the full texts. Finally, 7 studies (Liao et al., 2017; Liao et al., 2018; Banitalebi et al., 2020; Vasconcelos et al., 2016; Cunha et al., 2018; Huang et al., 2017; Lee et al., 2021) were selected for analysis. The detailed screening flowchart is shown in Figure 2.

### 3.2 Characteristics of the included studies

A total of seven RCTs were included in the meta-analysis of this study. The total number of participants across all studies was 303, with 162 in the experimental group and 141 in the control group, all aged between 60 and 90 years. Body fat percentage (BF%) and skeletal muscle index (SMI) are the most commonly used indicators for distinguishing between obesity and sarcopenia. Bioelectrical impedance analysis (BIA), as a crucial tool for assessing body composition, is widely favored by researchers. Five studies (Liao et al., 2017; Liao et al., 2018; Banitalebi et al., 2020; Huang et al., 2017; Lee et al., 2021) used resistance band training as a form of RT. The majority of interventions implemented a training regimen of 3 days/week over a 12-week period, with the exception of one study (Vasconcelos et al., 2016) utilizing a 10-week protocol. All training sessions were supervised, and participants demonstrated good compliance throughout the interventions. The outcome measures involved several aspects: body composition [BF%, BMI, Bone mass density (BMD), Total skeletal muscle mass (TSM)] and physical function [Timed up and go test (TUG), 10-min walk test (10MWT), Timed chair rise test (TCR)]. General information about these studies and their main characteristics are listed in Table 1.

### 3.3 Effect of RT on body composition in older females with SO

Six studies (Liao et al., 2017; Liao et al., 2018; Banitalebi et al., 2020; Cunha et al., 2018; Huang et al., 2017; Lee et al., 2021) investigated the impact of RT on BF% in older females with SO. The fixed-effects model indicated moderate heterogeneity (*p* = 0.07, *I*^2^ = 51%). As a result, we applied a random-effects model. The meta-analysis results demonstrated that the BF% in the RT group was significantly lower than in the control group (WMD = −2.83, 95% CI: −4.55 to −1.12, *p* = 0.001), suggesting that RT can effectively reduce BF% in older females with SO.

**Figure 3:**
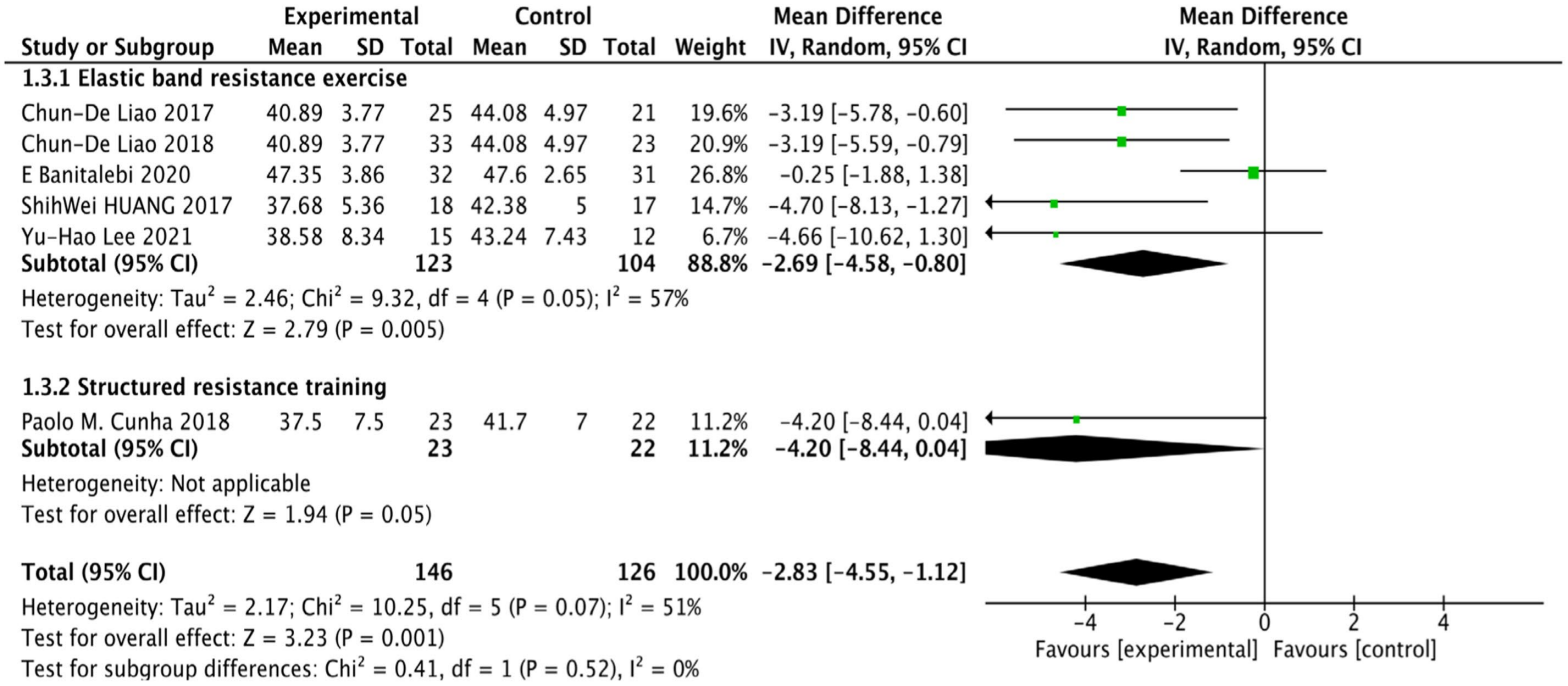
Forest plot comparing BF% between the RT group and the control group in older females with SO.

Subgroup analyses stratified by the type of RT revealed that both elastic band resistance exercise and structured resistance training significantly reduced BF% in patients [WMD = −2.69, 95% CI: −4.58 to −0.80, *p* = 0.005, WMD = −4.20, 95% CI: −8.44 to −0.04, *p* = 0.05], as illustrated in Figure 3.

**Figure 4:**
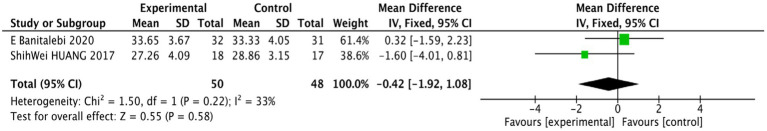
Forest plot comparing BMI between the RT group and the control group in older females with SO.

The two included studies (Banitalebi et al., 2020; Huang et al., 2017) exhibited mild heterogeneity (*p* = 0.22, *I*^2^ = 33%), suggesting good homogeneity and supporting reliable conclusions. Consequently, a fixed-effects model was employed for the analysis. The analysis revealed no statistically significant difference in BMI between the RT group and the control group in older females with SO (WMD = −0.42, 95% CI: −1.92 to 1.08, *p* = 0.58), as illustrated in Figure 4.

**Figure 5:**
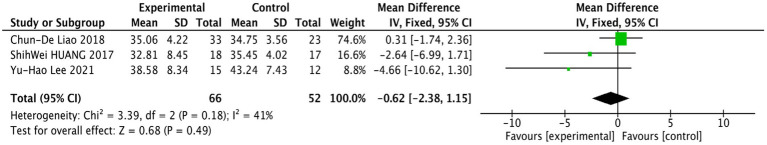
Forest plot comparing TSM between the RT group and the control group in older females with SO.

TSM: The three studies on TSM (Liao et al., 2017; Huang et al., 2017; Lee et al., 2021) showed mild heterogeneity (*p* = 0.18, *I*^2^ = 41%). Therefore, a fixed-effects model was applied for the analysis. The results showed no statistically significant difference in the TSM index between the RT group and the control group among older females with SO (WMD = −0.62, 95% CI: −2.38 to 1.15, *p* = 0.49), as illustrated in Figure 5.

**Figure 6:**
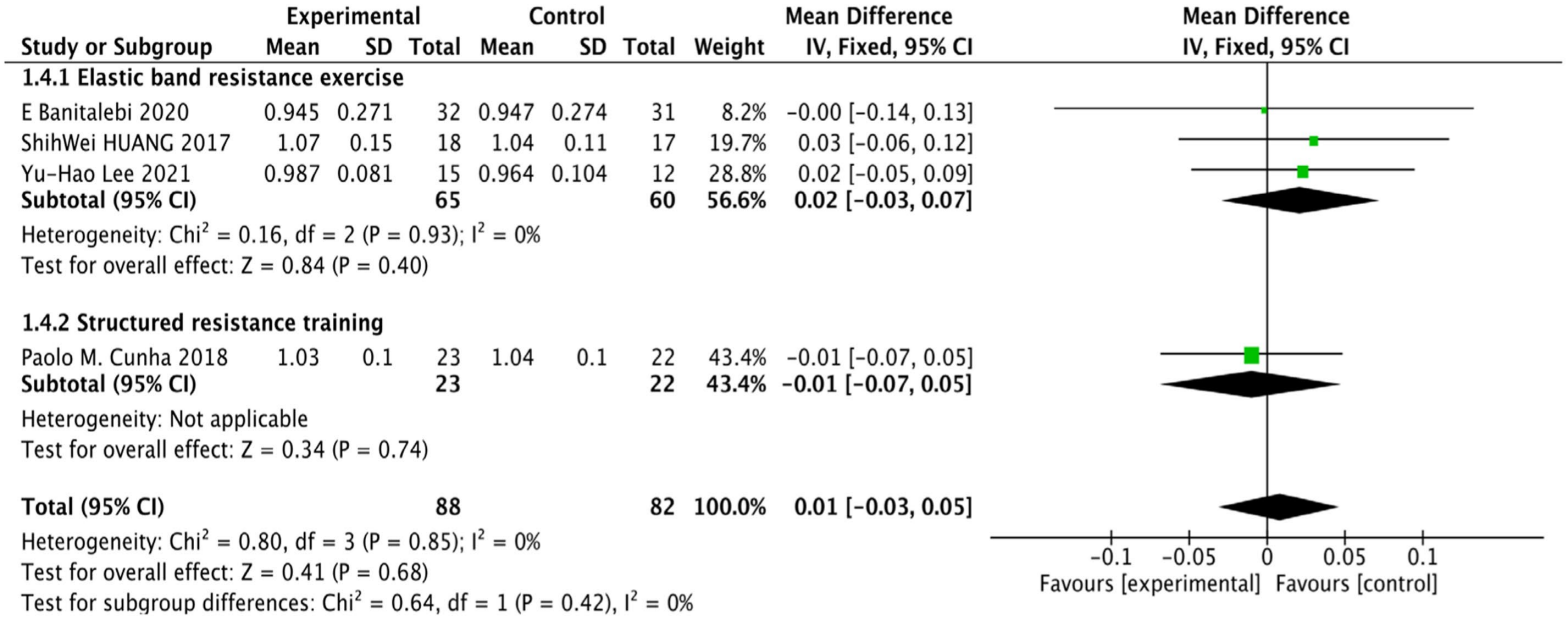
Forest plot comparing BMD between the RT group and the control group in older females with SO.

The four studies on BMD (Banitalebi et al., 2020; Cunha et al., 2018; Huang et al., 2017; Lee et al., 2021) showed no heterogeneity, demonstrating strong homogeneity (*p* = 0.85, *I*^2^ = 0%), indicating that the conclusions are reliable. The analysis revealed no statistically significant difference in the BMD index between the RT group and the control group among older females with SO (WMD = 0.01, 95% CI: −0.03 to 0.05, *p* = 0.68), as illustrated in Figure 6.

Subgroup analyses stratified by RT modalities revealed that neither elastic band resistance exercise nor structured resistance training demonstrated statistically significant effects on BMD in older females with SO (WMD = 0.02, 95% CI: −0.03 to 0.07, *p* = 0.40, WMD = −0.01, 95% CI: −0.07 to 0.05, *p* = 0.75), as illustrated in Figure 6.

#### 3.3.1 Effect of RT on physical function in older females with SO

**Figure 7:**
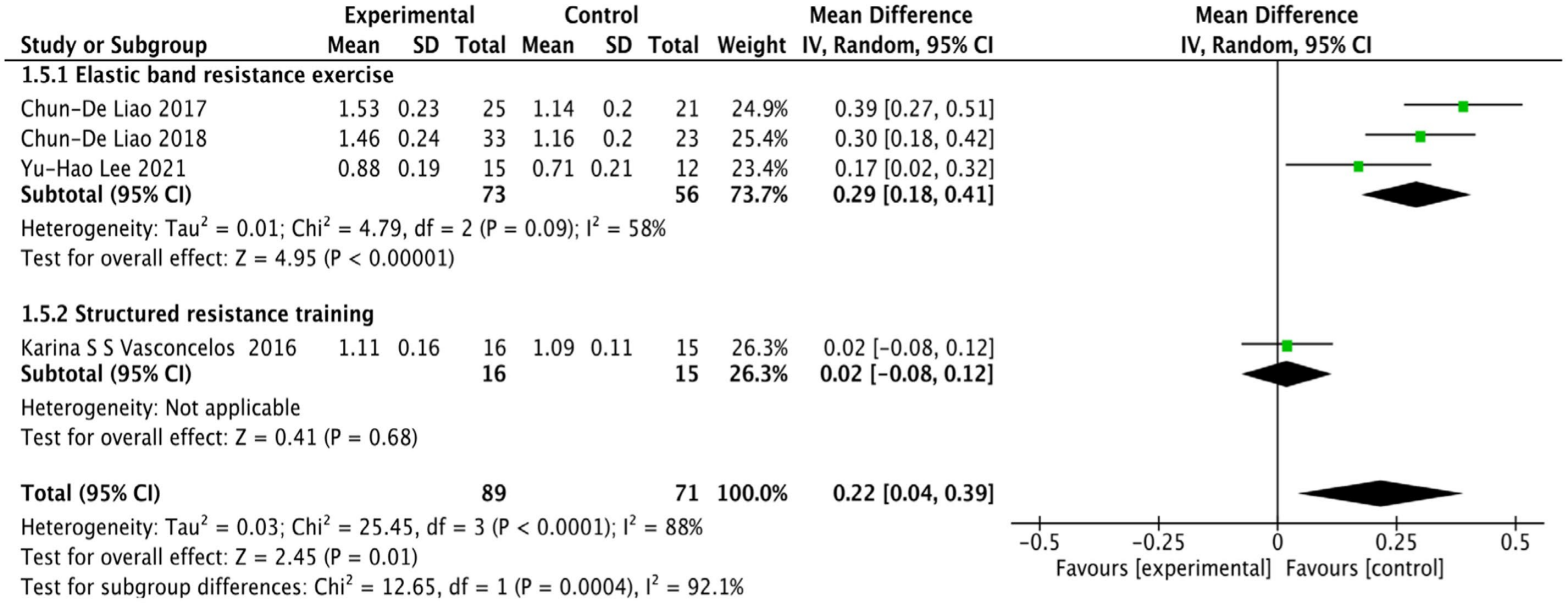
Forest plot comparing 10WMT between the RT group and the control group in older females with SO.

The four studies on 10WMT (Liao et al., 2017; Liao et al., 2018; Vasconcelos et al., 2016; Lee et al., 2021) showed significant heterogeneity (*p* = 0.0001, *I*^2^ = 88%), so a random-effects model was used for analysis. The results showed a statistically significant difference in the 10WMT index between the RT group and the control group among older females with SO (WMD = 0.22, 95% CI: 0.04 to 0.39, *p* = 0.01). This indicates that RT can effectively improve the physical function of older females with SO, as shown in Figure 7.

Subgroup analyses stratified by RT modalities demonstrated that elastic band resistance exercise significantly improved the 10MWT performance in older females with SO (WMD = 0.29, 95% CI: 0.18 to 0.41, *p* < 0.00001), whereas structured resistance training showed no statistically significant effect on 10MWT in this population (WMD = 0.02, 95% CI: −0.08 to 0.12, *p* = 0.68), as illustrated in Figure 7.

**Figure 8:**
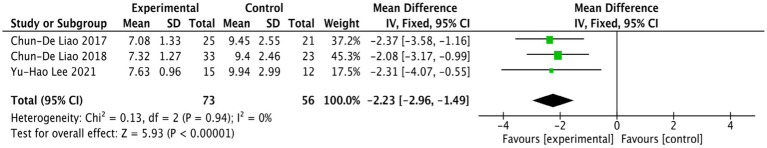
Forest plot comparing TUG between the RT group and the control group in older females with SO.

The three studies on TUG (Liao et al., 2017; Liao et al., 2018; Lee et al., 2021) showed no heterogeneity, demonstrating strong homogeneity (*p* = 0.94, *I*^2^ = 0.0%), indicating that the conclusions are reliable. The analysis showed a statistically significant difference in the TUG index between the RT group and the control group among older females with SO (WMD = −2.23, 95% CI: −2.96 to −1.49, *p* = 0.00001), as illustrated in Figure 8.

**Figure 9:**
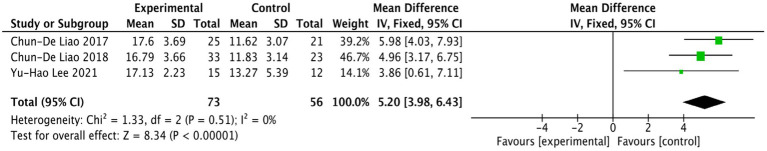
Forest plot comparing TCR between the RT group and the control group in older females with SO.

The three studies on TCR (Liao et al., 2017; Liao et al., 2018; Lee et al., 2021) showed no heterogeneity, demonstrating strong homogeneity (*p* = 0.51, *I*^2^ = 0.0%), indicating that the conclusions are reliable. The analysis showed a statistically significant difference in the TCR index between the RT group and the control group among older females with SO (WMD = 5.20, 95% CI: 3.98 to 6.43, *p* = 0.00001), as illustrated in Figure 9.

#### 3.3.2 Sensitivity analysis

**Figure 10:**
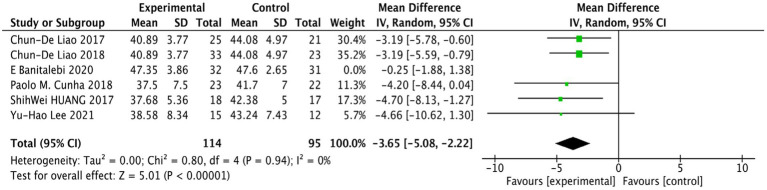
Sensitivity analysis of the outcome measure: BF%.

The sensitivity analysis results showed that Banitalebi et al. (2020) had the greatest influence on the heterogeneity of the included studies on BF%. After excluding this study, no heterogeneity was found among the remaining studies (*p* = 0.94, *I*^2^ = 0%). A full review revealed that the source of heterogeneity was due to this study’s outcomes being contrary to the others, specifically showing that RT had no effect on improving BF% in older females with SO. The remaining results were consistent, and the conclusions were reliable. The specific results are shown in Figure 10.

#### 3.3.3 Results of description analysis

Due to the high heterogeneity of the 10WMT metric, which could not be explored through subgroup analysis, we conducted a descriptive analysis. Among the four included studies (Liao et al., 2017; Liao et al., 2018; Vasconcelos et al., 2016; Lee et al., 2021), the results of Chun-De Liao 2017, Chun-De Liao 2018, and Yu-Hao Lee (Liao et al., 2017; Liao et al., 2018; Lee et al., 2021) were consistent. RT was found to improve the 10WMT scores in older females with SO, while Vasconcelos KS’ (Vasconcelos et al., 2016) study showed that RT did not improve the 10WMT scores in SO older females, nor did it enhance related physical function indicators such as strength and fatigue index.

## 4 Discussion

### 4.1 Overall findings

This study is the first to conduct a meta-analysis of existing RCTs examining the effects of RT on SO in older females. The meta-analysis searched most databases and applied strict inclusion and exclusion criteria. Overall, compared to the control group, the RT group showed significant training effects on BF%, 10WMT test, TUG test, and TCR test. However, there was no significant effect on BMI, TSM, or BMD. In addition, although the study results were influenced by training methods, duration, intensity, and sample size, the good quality of the included studies provides sufficient grounds to support the necessity of exercise interventions for older females with SO in the future.

#### 4.1.1 Body composition

Altering body composition through RT in older females with SO is challenging, yet our study demonstrates that RT can effectively reduce BF% in these patients, consistent with the meta-analysis by Liu and Lee (2024). A plausible explanation is that exercise not only increases mitochondrial enzyme activity and lipid oxidation within muscle cells (Takemura et al., 2024), but also reduces fat content through endocrine pathways. Animal studies suggest that RT increases the reactivity of *α*-ketoglutarate (AKG), an intermediate in the tricarboxylic acid (TCA) cycle (Leibowitz et al., 2012). As a metabolite responsive to exercise, AKG significantly stimulates the release of hormone E from the adrenal medulla, which in turn activates thermogenesis and lipolysis in fat cells. Further mechanistic studies revealed that OXGR1, an orphan G protein-coupled receptor for AKG, is highly expressed in the adrenal glands (Yuan et al., 2022). Researchers found that AKG did not promote lipolysis in OXGR1-deficient mice, indicating that OXGR1 plays a key role in mediating AKG’s metabolic effects (Yuan et al., 2020), offering new insights into how RT reduces BF%. Therefore, we strongly recommend exercise, particularly resistance training, to reduce body fat.

Theoretically, RT may enhance bone mineral density BMD and TSM. However, our meta-analysis revealed that while both BMD and TSM increased post-intervention in the RT group compared to controls, no statistically significant between-group differences were observed. We discuss these findings separately to elucidate potential explanations. Regarding BMD outcomes, several factors warrant consideration. First, the limitations of measurement tools: although DXA is widely used for body composition assessment, it cannot detect subtle structural changes in bone architecture, necessitating more precise methods to comprehensively evaluate bone mineral status (Chuang et al., 2024). Second, the differential effects of weight-bearing versus non-weight-bearing skeletal sites are critical. Observations indicate that the radius—a non-weight-bearing site—exhibits more pronounced declines in BMD, structural integrity, and biomechanical strength compared to the tibia. Consequently, even moderate mechanical stimuli may induce measurable BMD improvements in the radius (Wochna et al., 2022). In contrast, the tibia is continuously exposed to ground reaction forces during daily activities. Thus, whether conventional RT or elastic band training over 12 weeks—despite improving muscle strength—delivers sufficient osteogenic stimuli to effectively modulate bone metabolism and remodeling remains debatable (Beck, 2009). Notably, most included studies employed moderate-intensity RT protocols, necessitating cautious interpretation of these results. Future research should prioritize personalized, progressive, and long-term resistance training protocols tailored to individual patient profiles, incorporating moderate-to-high intensity (70–80% of 1RM) strength training to optimize skeletal adaptation. Such protocols may better address the mechanostat threshold required for osteogenic responses, particularly in weight-bearing regions.

The findings pertaining to TSM may be attributed to several potential factors. First, older females with SO may have a diminished response to exercise. A study suggests that older individuals undergoing RT experience reduced ribosome biogenesis, which may be a potential mechanism driving age-related muscle mass decline induced by RT (Stec et al., 2015). Reduced insulin sensitivity may be another reason for the weakened response to exercise in older females with SO, as it has been shown to decrease muscle protein breakdown and stimulate muscle protein synthesis (Tardif et al., 2014). Second, factors such as age and obesity may interfere with the metabolism of older females with SO. Lower training durations and intensities may not sufficiently stimulate osteogenesis or muscle synthesis. Therefore, it is recommended to continue RT for at least 6 months to observe more significant improvements in TSM in older populations (Frimel et al., 2008). Thirdly, age-related anabolic resistance is influenced by multiple factors including digestive efficiency, nutrient absorption, synthesis of anabolic signaling proteins, visceral amino acid sequestration, physical activity levels, and postprandial amino acid availability/delivery (Burd et al., 2013). It has been proposed that enhanced protein supplementation during RT may more effectively augment skeletal muscle mass (Rondanelli et al., 2016). However, the studies included in this analysis solely examined RT interventions without incorporating dietary protein variables as a controlled parameter. This single-component approach might limit the efficacy in improving TSM among older women with SO. Finally, most included studies utilized elastic bands as the primary RT modality. While elastic band training demonstrates high acceptability in older populations due to its simplicity, portability, low spatial requirements, and cost-effectiveness, this design raises methodological concerns (Hernandez-Martinez et al., 2024). The emphasis on elastic resistance may inadvertently shift training focus from strength development to endurance adaptation, potentially altering study outcomes. Furthermore, the inherent elastic resistance properties complicate precise load quantification and exhibit lower activation efficiency of fast-twitch muscle fibers, creating physiological bottlenecks in strength gains and muscle hypertrophy (Melchiorri and Rainoldi, 2011). More structured RT can better recruit fast-contracting fibers, improve neuromuscular adaptations, and increase muscle mass and strength through scientifically designed periodized loads, task-specific movement selection, and precise speed control (Sterczala et al., 2024). Consequently, future clinical trials should consider implementing higher-intensity RT protocols, which - when progressively administered under appropriate supervision—have demonstrated safety and tolerability while potentially yielding greater functional improvements in this population.

Furthermore, the results of this study demonstrated that while RT reduced BMI in older females with SO, no statistically significant between-group differences were observed. Considering that the study did not control participants’ diets or include nutritional education and assessment, variations in dietary intake may have contributed to the inaccuracy of the results. Testosterone, as an anabolic androgenic hormone, also plays a role in muscle synthesis (Zuo et al., 2024). In general, testosterone levels in women gradually decline and then rapidly decrease later in life (Vingren et al., 2010). However, research on the testosterone response to RT programs in women remains inconclusive. Furthermore, considering that the results of this study are based on only two studies, we should interpret this indicator cautiously to ensure the validity of the study’s findings. Future research should set stricter inclusion and exclusion criteria or combine RT with aerobic exercise and dietary adjustments to fully explore the impact of RT on BMI in older females with SO.

#### 4.1.2 Physical function

Older females with SO are often accompanied by an increased risk of falls and a decline in balance. We found that RT interventions significantly improved all analyzed physical function measures (*p* < 0.05). Although the assessment scale used in this study is somewhat subjective, we believe these results have important implications for future clinical research.

Dynamic balance, mobility, and gait speed are clinical markers of physical function in older populations, and these can be assessed using the 10WMT test and TUG scales. Muscle weakness and reduced gait speed are strong predictors of functional impairment, which can lead to immobility and limited ability to perform activities of daily living (ADL) (Marsh et al., 2011; Kojima, 2018). Lower ADL function may also result in higher mortality rates (Stineman et al., 2012). The 10WMT test and TUG test are commonly used to assess functional parameters in older care. As sensitive predictors of recurrent falls, they are closely related to the daily life of SO patients (Waterval et al., 2023). Therefore, improving 10WMT test and TUG scores is of great significance for the physical recovery of SO patients. SO typically induces progressive physical capacity deterioration in affected individuals. RT, recognized as an optimal modality for augmenting muscle strength, has demonstrated efficacy in counteracting SO-related functional decline and enhancing physical performance among older females with this condition.

The TCR test not only assesses a person’s balance but also reflects their strength (Kilpi et al., 2022). In our study, RT also demonstrated considerable clinical value. The potential mechanism is that RT improves neuro muscular parameters, including better motor unit recruitment, intra and inter-muscular coordination, and firing frequency (Stoever et al., 2018; Egan and Sharples, 2023). As muscle strength and physical function increase, older individuals can enhance their quality of life and achieve greater functional independence (Sidney et al., 2023). Therefore, older females can benefit from RT, counteracting the harmful effects caused by SO.

## 5 Conclusion

In conclusion, RT has demonstrated efficacy in improving physical function and specific body composition parameters (BF%) in older females with SO. Furthermore, although RT demonstrated measurable effects on BMI, TSM, and BMD in this population, the between-group comparisons failed to reach statistical significance.

Of course, we must also address the limitations of this study. First, this study included only seven clinical trials, with a small sample size, which reduces the generalizability and reliability of the findings. Second, in an effort to increase sample size and the reliability of outcomes, this study included older females with osteosarcopenic obesity. Although this population includes individuals with SO alone, the changes in bone mass and more complex pathological mechanisms in these patients suggest that future studies should consider separating these groups. Third, this study only examined the effects of RT alone. However, it is worth considering that RT alone may not be sufficient to significantly improve relevant outcomes. A meta-analysis has shown that combining protein supplementation with RT produces better results in both body composition and functional improvement (Liao et al., 2017). Future research should focus on combined interventions to further explore their effectiveness. Finally, the outcome measures included in this study were limited. Due to differences in outcome measures across studies, some could not be included in the meta-analysis. As a result, not all body composition and physical function (e.g., leptin, 5-time sit-to-stand test [5STS], isometric handgrip strength [IHG], etc.) were explored.

Nevertheless, we believe that the meta-analysis still holds significant value. Through our literature review, we found a general lack of randomized controlled trials on the application of RT in older females with SO. Although we included only seven clinical trials in the meta-analysis, the results provide valuable direction for the development of future treatment protocols. In the future, we should focus our research on the following aspects: (1) Precise population stratification and expanding sample diversity, conducting multi-center RCTs, and strictly expanding the sample size according to inclusion and exclusion criteria. Grouping by bone density and inflammatory marker levels to assess the differential effects of RT. (2) Optimization and validation of combined intervention strategies, designing a three-arm trial to compare the synergistic effects of pure RT, RT combined with protein supplementation, and RT + protein + vitamin D3. (3) Standardized evaluation system and expanded outcome indicators, adopting multimodal evaluation methods (imaging and functional assessments), adding molecular markers of SO, such as serum GDF-15 and osteocalcin. (4) Developing personalized exercise programs, grading exercise intensity according to baseline muscle strength and optimizing data based on patient compliance. (5) Establishing long-term safety and cost-effectiveness evaluations, following up on data for the next 2 years and conducting health economics analysis.

## Data Availability

The original contributions presented in the study are included in the article/Supplementary material, further inquiries can be directed to the corresponding authors.

## References

[ref1] Arora N. K. Donath L. Owen P. J. Miller C. T. Kaczorowski S. Saueressig T. . (2024). DOSage of exercise for chronic low back pain disorders (DOSE): protocol for a systematic review with dose-response network meta-analysis. BMJ Open Sport Exerc. Med. 10:e002108. doi: 10.1136/bmjsem-2024-002108, PMID: 39161554 PMC11331831

[ref2] Assyov Y. Nedeva I. Spassov B. Gerganova A. Velikov T. Kamenov Z. . (2024). Nutritional management and physical activity in the treatment of Sarcopenic obesity: a review of the literature. Nutrients 16:2560. doi: 10.3390/nu16152560, PMID: 39125439 PMC11314398

[ref3] Banitalebi E. Faramarzi M. Ghahfarokhi M. M. SavariNikoo F. Soltani N. Bahramzadeh A. (2020). Osteosarcopenic obesity markers following elastic band resistance training: a randomized controlled trial. Exp. Gerontol. 135:110884. doi: 10.1016/j.exger.2020.110884, PMID: 32092502

[ref4] Barbagallo F. Cucinella L. Tiranini L. Chedraui P. Calogero A. E. Nappi R. E. (2024). Obesity and sexual health: focus on postmenopausal women. Climacteric 27, 122–136. doi: 10.1080/13697137.2024.2302429, PMID: 38251874

[ref5] Beck B. R. (2009). Muscle forces or gravity--what predominates mechanical loading on bone? Introduction. Med. Sci. Sports Exerc. 41, 2033–2036. doi: 10.1249/MSS.0b013e3181a8c4b6 19812514

[ref6] Burd N. A. Gorissen S. H. van Loon L. J. (2013). Anabolic resistance of muscle protein synthesis with aging. Exerc. Sport Sci. Rev. 41, 169–173. doi: 10.1097/JES.0b013e318292f3d5, PMID: 23558692

[ref7] Cashin A. G. McAuley J. H. (2020). Clinimetrics: physiotherapy evidence database (PEDro) scale. J. Physiother. 66:59. doi: 10.1016/j.jphys.2019.08.005, PMID: 31521549

[ref8] Chuang C. L. Lai C. L. Huang A. C. Su P. H. Chu L. P. Hsieh K. C. . (2024). Comparison of whole body bone mineral density measurements between dual energy X-ray absorptiometry and novel bioelectrical impedance analysis. Sci. Rep. 14:29127. doi: 10.1038/s41598-024-80721-7, PMID: 39582046 PMC11586393

[ref9] Cunha P. M. Ribeiro A. S. Tomeleri C. M. Schoenfeld B. J. Silva A. M. Souza M. F. . (2018). The effects of resistance training volume on osteosarcopenic obesity in older women. J. Sports Sci. 36, 1564–1571. doi: 10.1080/02640414.2017.1403413, PMID: 29125017

[ref10] Debes W. A. Sadaqa M. Németh Z. Aldardour A. Prémusz V. Hock M. (2024). Effect of resistance exercise on body composition and physical function in older women with Sarcopenic obesity-a systematic review with narrative synthesis. J. Clin. Med. 13:441. doi: 10.3390/jcm13020441, PMID: 38256574 PMC10817090

[ref11] Dominguez L. J. Barbagallo M. (2007). The cardiometabolic syndrome and sarcopenic obesity in older persons. J. Cardiometab. Syndr. 2, 183–189. doi: 10.1111/j.1559-4564.2007.06673.x, PMID: 17786082

[ref12] Egan B. Sharples A. P. (2023). Molecular responses to acute exercise and their relevance for adaptations in skeletal muscle to exercise training. Physiol. Rev. 103, 2057–2170. doi: 10.1152/physrev.00054.2021, PMID: 36395350

[ref13] Frimel T. N. Sinacore D. R. Villareal D. T. (2008). Exercise attenuates the weight-loss-induced reduction in muscle mass in frail obese older adults. Med. Sci. Sports Exerc. 40, 1213–1219. doi: 10.1249/MSS.0b013e31816a85ce, PMID: 18580399 PMC2650077

[ref14] Gadelha A. B. Paiva F. M. Gauche R. de Oliveira R. J. Lima R. M. (2016). Effects of resistance training on sarcopenic obesity index in older women: a randomized controlled trial. Arch. Gerontol. Geriatr. 65, 168–173. doi: 10.1016/j.archger.2016.03.017, PMID: 27057600

[ref15] Gavras A. Batsis J. A. (2024). Medical weight loss in older persons with obesity. Clin. Obes. 14:e12684. doi: 10.1111/cob.12684, PMID: 38924367 PMC11570349

[ref16] Ghiotto L. Muollo V. Tatangelo T. Schena F. Rossi A. P. (2022). Exercise and physical performance in older adults with sarcopenic obesity: a systematic review. Front. Endocrinol. 13:913953. doi: 10.3389/fendo.2022.913953, PMID: 35966077 PMC9366852

[ref17] Hernandez-Martinez J. Cid-Calfucura I. Chiguay C. Weinberger M. Delgado-Floody P. Muñoz-Vásquez C. . (2024). Effects of elastic band training on body composition and physical performance in older people: a systematic review with meta-analysis. Exp. Gerontol. 196:112553. doi: 10.1016/j.exger.2024.112553, PMID: 39197674

[ref18] Huang S. W. Ku J. W. Lin L. F. Liao C. D. Chou L. C. Liou T. H. (2017). Body composition influenced by progressive elastic band resistance exercise of sarcopenic obesity older females: a pilot randomized controlled trial. Eur. J. Phys. Rehabil. Med. 53, 556–563. doi: 10.23736/S1973-9087.17.04443-4, PMID: 28084062

[ref19] Jang W. Kim H. (2023). Association of socioeconomic factors and dietary intake with sarcopenic obesity in the Korean older population. Asia Pac. J. Clin. Nutr. 32, 348–355. doi: 10.6133/apjcn.202309_32(3).0006, PMID: 37789655 PMC11090395

[ref20] Javed A. A. Aljied R. Allison D. J. Anderson L. N. Ma J. Raina P. (2020). Body mass index and all-cause mortality in older adults: a scoping review of observational studies. Obes. Rev. 21:e13035. doi: 10.1111/obr.13035, PMID: 32319198

[ref22] Kilpi F. Soares A. G. Clayton G. L. Fraser A. Welsh P. Sattar N. . (2022). Changes in women's physical function in mid-life by reproductive age and hormones: a longitudinal study. BMC Womens Health 22:473. doi: 10.1186/s12905-022-02070-9, PMID: 36434722 PMC9700972

[ref23] Kojima G. (2018). Quick and simple FRAIL scale predicts incident activities of daily living (ADL) and instrumental ADL (IADL) disabilities: a systematic review and Meta-analysis. J. Am. Med. Dir. Assoc. 19, 1063–1068. doi: 10.1016/j.jamda.2018.07.019, PMID: 30206033

[ref24] Lee K. (2024). Trends in prevalence of overweight and obesity, self-perceived overweight or obesity, and weight loss efforts among older adults in South Korea, 2005-2021. Prev. Med. 180:107854. doi: 10.1016/j.ypmed.2024.107854, PMID: 38211800

[ref25] Lee Y. H. Lee P. H. Lin L. F. Liao C. D. Liou T. H. Huang S. W. (2021). Effects of progressive elastic band resistance exercise for aged osteosarcopenic adiposity women. Exp. Gerontol. 147:111272. doi: 10.1016/j.exger.2021.111272, PMID: 33549820

[ref26] Lee J. Lim B. O. Byeon J. Y. Seok R. (2024). Effects of participation in an eight-week, online video body-weight resistance training on cognitive function and physical fitness in older adults: a randomized control trial. Geriatr. Nurs. 58, 98–103. doi: 10.1016/j.gerinurse.2024.05.001, PMID: 38788559

[ref27] Leibowitz A. Klin Y. Gruenbaum B. F. Gruenbaum S. E. Kuts R. Dubilet M. . (2012). Effects of strong physical exercise on blood glutamate and its metabolite 2-ketoglutarate levels in healthy volunteers. Acta Neurobiol. Exp. 72, 385–396. doi: 10.55782/ane-2012-1910, PMID: 23377269

[ref28] Liao C. D. Tsauo J. Y. Huang S. W. Ku J. W. Hsiao D. J. Liou T. H. (2018). Effects of elastic band exercise on lean mass and physical capacity in older women with sarcopenic obesity: a randomized controlled trial. Sci. Rep. 8:2317. doi: 10.1038/s41598-018-20677-7, PMID: 29396436 PMC5797161

[ref29] Liao C. D. Tsauo J. Y. Lin L. F. Huang S. W. Ku J. W. Chou L. C. . (2017). Effects of elastic resistance exercise on body composition and physical capacity in older women with sarcopenic obesity: a CONSORT-compliant prospective randomized controlled trial. Medicine 96:e7115. doi: 10.1097/MD.0000000000007115, PMID: 28591061 PMC5466239

[ref30] Liao C. D. Tsauo J. Y. Wu Y. T. Cheng C. P. Chen H. C. Huang Y. C. . (2017). Effects of protein supplementation combined with resistance exercise on body composition and physical function in older adults: a systematic review and meta-analysis. Am. J. Clin. Nutr. 106, 1078–1091. doi: 10.3945/ajcn.116.143594, PMID: 28814401

[ref31] Liu X. M. Chan H. C. Ding G. L. Cai J. Song Y. Wang T. T. . (2015). FSH regulates fat accumulation and redistribution in aging through the Gαi/Ca(2+)/CREB pathway. Aging Cell 14, 409–420. doi: 10.1111/acel.12331, PMID: 25754247 PMC4406670

[ref32] Liu H. W. Lee O. K. (2024). Effects of resistance training with elastic bands on bone mineral density, body composition, and osteosarcopenic obesity in older females: a meta-analysis. J. Orthop. 53, 168–175. doi: 10.1016/j.jor.2024.03.039 38633989 PMC11018988

[ref33] Marsh A. P. Rejeski W. J. Espeland M. A. Miller M. E. Church T. S. Fielding R. A. . (2011). Muscle strength and BMI as predictors of major mobility disability in the lifestyle interventions and Independence for elders pilot (LIFE-P). J. Gerontol. A Biol. Sci. Med. Sci. 66, 1376–1383. doi: 10.1093/gerona/glr158 21975090 PMC3210962

[ref34] Melchiorri G. Rainoldi A. (2011). Muscle fatigue induced by two different resistances: elastic tubing versus weight machines. J. Electromyogr. Kinesiol. 21, 954–959. doi: 10.1016/j.jelekin.2011.07.015, PMID: 21920774

[ref35] Page M. J. McKenzie J. E. Bossuyt P. M. Boutron I. Hoffmann T. C. Mulrow C. D. . (2021). The PRISMA 2020 statement: an updated guideline for reporting systematic reviews. BMJ 372:n71. doi: 10.1136/bmj.n71, PMID: 33782057 PMC8005924

[ref36] Rondanelli M. Klersy C. Terracol G. Talluri J. Maugeri R. Guido D. . (2016). Whey protein, amino acids, and vitamin D supplementation with physical activity increases fat-free mass and strength, functionality, and quality of life and decreases inflammation in sarcopenic elderly. Am. J. Clin. Nutr. 103, 830–840. doi: 10.3945/ajcn.115.113357, PMID: 26864356

[ref37] Sidney A. S. Nunes E. A. Lim C. McKendry J. Phillips S. M. (2023). The health benefits of resistance exercise: beyond hypertrophy and big weights. Exerc. Sport Mov. 1:e00001. doi: 10.1249/ESM.0000000000000001

[ref38] Sinclair A. J. Laosa O. Antonio Carnicero J. Rodriguez-Mañas L. Álvarez-Bustos A. MIDFRAIL consortium (2024). Disability and quality of life measures in older frail and prefrail people with type 2 diabetes. The MIDFRAIL-study. Diabetes Res. Clin. Pract. 214:111797. doi: 10.1016/j.diabres.2024.111797, PMID: 39074514

[ref39] Stec M. J. Mayhew D. L. Bamman M. M. (2015). The effects of age and resistance loading on skeletal muscle ribosome biogenesis. J. Appl. Physiol. 119, 851–857. doi: 10.1152/japplphysiol.00489.2015, PMID: 26294750 PMC4747892

[ref40] Stenholm S. Rantanen T. Heliövaara M. Koskinen S. (2008). The mediating role of C-reactive protein and handgrip strength between obesity and walking limitation. J. Am. Geriatr. Soc. 56, 462–469. doi: 10.1111/j.1532-5415.2007.01567.x, PMID: 18179481

[ref41] Sterczala A. J. Rodriguez-Ortiz N. Feigel E. D. Krajewski K. T. Martin B. J. Sekel N. M. . (2024). Skeletal muscle adaptations to high-intensity, low-volume concurrent resistance and interval training in recreationally active men and women. Physiol. Rep. 12:e15953. doi: 10.14814/phy2.15953, PMID: 38490811 PMC10942853

[ref42] Sterne J. A. C. Savović J. Page M. J. Elbers R. G. Blencowe N. S. Boutron I. . (2019). RoB 2: a revised tool for assessing risk of bias in randomised trials. BMJ 366:l4898. Published 2019 Aug 28. doi: 10.1136/bmj.l4898, PMID: 31462531

[ref43] Stineman M. G. Xie D. Pan Q. Kurichi J. E. Zhang Z. Saliba D. . (2012). All-cause 1-, 5-, and 10-year mortality in elderly people according to activities of daily living stage. J. Am. Geriatr. Soc. 60, 485–492. doi: 10.1111/j.1532-5415.2011.03867.x, PMID: 22352414 PMC3302958

[ref44] Stoever K. Heber A. Eichberg S. Brixius K. (2018). Influences of resistance training on physical function in older, obese men and women with sarcopenia. J. Geriatr. Phys. Ther. 41, 20–27. doi: 10.1519/JPT.0000000000000105, PMID: 27824658

[ref45] Takemura A. Matsunaga Y. Shinya T. Matta H. (2024). Differential mitochondrial adaptation of the slow and fast skeletal muscles by endurance running exercise in streptozotocin-induced diabetic mice. Physiol. Res. 73, 369–379. doi: 10.33549/physiolres.935183, PMID: 39027954 PMC11299777

[ref46] Tardif N. Salles J. Guillet C. Tordjman J. Reggio S. Landrier J. F. . (2014). Muscle ectopic fat deposition contributes to anabolic resistance in obese sarcopenic old rats through eIF2α activation. Aging Cell 13, 1001–1011. doi: 10.1111/acel.12263 25139155 PMC4326920

[ref47] Vasconcelos K. S. Dias J. M. Araújo M. C. Pinheiro A. C. Moreira B. S. Dias R. C. (2016). Effects of a progressive resistance exercise program with high-speed component on the physical function of older women with sarcopenic obesity: a randomized controlled trial. Braz. J. Phys. Ther. 20, 432–440. doi: 10.1590/bjpt-rbf.2014.0174 27410162 PMC5123261

[ref48] Villareal D. T. Banks M. Siener C. Sinacore D. R. Klein S. (2004). Physical frailty and body composition in obese elderly men and women. Obes. Res. 12, 913–920. doi: 10.1038/oby.2004.111, PMID: 15229329

[ref49] Vingren J. L. Kraemer W. J. Ratamess N. A. Anderson J. M. Volek J. S. Maresh C. M. (2010). Testosterone physiology in resistance exercise and training: the up-stream regulatory elements. Sports Med. 40, 1037–1053. doi: 10.2165/11536910-000000000-00000 21058750

[ref50] Waterval N. F. J. Claassen C. M. van der Helm F. C. T. van der Kruk E. (2023). Predictability of fall risk assessments in community-dwelling older adults: a scoping review. Sensors 23:7686. doi: 10.3390/s23187686, PMID: 37765742 PMC10536675

[ref51] Winters-Stone K. M. Stoyles S. A. Dieckmann N. F. Eckstrom E. Luoh S. W. Horak F. B. . (2024). Can strength training or tai ji quan training reduce frailty in postmenopausal women treated with chemotherapy? A secondary data analysis of the GET FIT trial. J. Cancer Surviv. 18, 1179–1189. doi: 10.1007/s11764-024-01592-5, PMID: 38642204

[ref21] Wochna K. Ogurkowska M. Leszczyński P. Stemplewski R. Huta-Osiecka A. Błaszczyk A. . (2022). Nordic walking with an integrated resistance shock absorber ffects the femur strength and muscles torques in postmenopausal women. Sci Rep. 12, 20089. doi: 10.1038/s41598-022-24131-7, PMID: 36418455 PMC9684118

[ref52] Yuan Y. Xu P. Jiang Q. Cai X. Wang T. Peng W. . (2020). Exercise-induced α-ketoglutaric acid stimulates muscle hypertrophy and fat loss through OXGR1-dependent adrenal activation. EMBO J. 39:e103304. doi: 10.15252/embj.2019103304, PMID: 32104923 PMC7110140

[ref53] Yuan Y. Zhu C. Wang Y. Sun J. Feng J. Ma Z. . (2022). α-Ketoglutaric acid ameliorates hyperglycemia in diabetes by inhibiting hepatic gluconeogenesis via serpina1e signaling. Sci. Adv. 8:eabn2879. doi: 10.1126/sciadv.abn2879, PMID: 35507647 PMC9067931

[ref54] Zhang N. Qu X. Zhou H. Kang L. (2024). Mapping Knowledge Landscapes and emerging trends of Sarcopenic obesity in older adults: a bibliometric analysis from 2004 to 2023. Cureus 16:e62300. doi: 10.7759/cureus.62300 38873392 PMC11170931

[ref55] Zuo X. Bai H. J. Zhao Q. L. Zhang S. H. Zhao X. Feng X. Z. (2024). 17β-Trenbolone exposure enhances muscle activity and exacerbates Parkinson's disease progression in male mice. Mol. Neurobiol. 62, 3053–3066. doi: 10.1007/s12035-024-04455-3, PMID: 39222261

