# Supplementary material for: Effect of resistance training on body composition and physical function in older females with sarcopenic obesity—a systematic review and meta-analysis of randomized controlled trials
Source: Front Aging Neurosci. 2025 Apr 30;17:1495218. doi: 10.3389/fnagi.2025.1495218 (PMC12075210; doi:10.3389/fnagi.2025.1495218)
Supplement: Supplementary file 1 [file Supplementary_file_2.docx]

**Effect of Resistance training on Body Composition and Physical Function in Eldely Women with Sarcopenic Obesity— a systematic review and meta-analysis of controlled clinical trials Search Strategy**

| Database | Search strategy |
| --- | --- |
| Pubmed | #1 ( (((("Sarcopenia"[Mesh]) OR ("Muscular Atrophy"[Mesh])) OR ("Muscle Weakness"[Mesh])) OR (Sarcopenia[Title/Abstract])) OR (sarcopenic[Title/Abstract]) ) OR (Sarcopeni*[Title/Abstract])  #2 ((((((("Obesity"[Mesh]) OR ("Overweight"[Mesh])) OR (obese[Title/Abstract])) OR (obesity[Title/Abstract])) OR (obestic[Title/Abstract])) OR (overweight[Title/Abstract])) OR (obes*[Title/Abstract])) OR (adiposity[Title/Abstract])  #3 (((((((((((((((((((((((((((((((((("Weight Lifting"[Mesh])) OR ("Resistance Training"[Mesh])) OR (Resistance Training[Title/Abstract])) OR (resistance exercise[Title/Abstract])) OR (Training, Resistance[Title/Abstract])) OR (Strength Training[Title/Abstract])) OR (Training, Strength[Title/Abstract])) OR (Weight-Lifting Strengthening Program[Title/Abstract])) OR (Strengthening Program, Weight-Lifting[Title/Abstract])) OR (Strengthening Programs, Weight-Lifting[Title/Abstract])) OR (Weight Lifting Strengthening Program[Title/Abstract])) OR (Weight-Lifting Strengthening Programs[Title/Abstract])) OR (Weight-Lifting Exercise Program[Title/Abstract])) OR (Exercise Program, Weight-Lifting[Title/Abstract])) OR (Exercise Programs, Weight-Lifting[Title/Abstract])) OR (Weight Lifting Exercise Program[Title/Abstract])) OR (Weight-Lifting Exercise Programs[Title/Abstract])) OR (Weight-Bearing Strengthening Program[Title/Abstract])) OR (Strengthening Program, Weight-Bearing[Title/Abstract])) OR (Strengthening Programs, Weight-Bearing[Title/Abstract])) OR (Weight Bearing Strengthening Program[Title/Abstract])) OR (Weight-Bearing Strengthening Programs[Title/Abstract])) OR (Weight-Bearing Exercise Program[Title/Abstract])) OR (Exercise Program, Weight-Bearing[Title/Abstract])) OR (Exercise Programs, Weight-Bearing[Title/Abstract])) OR (Weight Bearing Exercise Program[Title/Abstract])) OR (Weight-Bearing Exercise Programs[Title/Abstract])) OR (Lifting, Weight[Title/Abstract])) OR (Liftings, Weight[Title/Abstract])) OR (Weight Liftings[Title/Abstract])) OR (elastic band[Title/Abstract])) OR (body weight training[Title/Abstract])) OR (strengthening exercise[Title/Abstract])) OR (strength exercise[Title/Abstract])  #4(((("Female"[Mesh]) OR ("Women"[Mesh])) OR (female[Title/Abstract])) OR (woman[Title/Abstract])) OR (women[Title/Abstract])  #5 (((((((((((((((("Single-Blind Method"[Mesh]) OR ("Double-Blind Method"[Mesh])) OR ("Randomized Controlled Trials as Topic"[Mesh])) OR (Randomized Controlled Trial[Publication Type])) OR ("Intention to Treat Analysis"[Mesh])) OR ("Controlled Clinical Trials as Topic"[Mesh])) OR ("Clinical Trials as Topic"[Mesh])) OR (Clinical Trial[Publication Type])) OR (randomized controlled trial[Publication Type])) OR (random*[Title/Abstract])) OR (allocation[Title/Abstract])) OR (random allocation[Title/Abstract])) OR (placebo[Title/Abstract])) OR (single blind[Title/Abstract])) OR (double blind[Title/Abstract])) OR (randomized controlled trial*[Title/Abstract])) OR (RCT[Title/Abstract])  #6 #1 AND #2 AND #3 AND #4 AND #5 |
| Web of science | #1=((((TS=(Sarcopenia)) OR TS=(sarcopenic)) OR TS=(Muscular Atrophy)) OR TS=(Muscle Weakness)) OR TS=(Sarcopeni*) and Preprint Citation Index (Exclude – Database)  #2=(((((TS=(obese)) OR TS=(obesity)) OR TS=(obestic)) OR TS=(overweight)) OR TS=(obes*)) OR TS=(adiposity) and Preprint Citation Index (Exclude – Database)  #3=(((((((((((((TS=(Weight Lifting)) OR TS=(Resistance Training)) OR TS=(resistance exercise)) OR TS=(Strength Training)) OR TS=(Weight-Lifting Strengthening Program)) OR TS=(Weight-Lifting Exercise Program)) OR TS=(Weight-Bearing Strengthening Program)) OR TS=(Weight Bearing Exercise Program)) OR TS=(Liftings, Weight)) OR TS=(Lifting, Weight)) OR TS=(elastic band)) OR TS=(body weight training)) OR TS=(strengthening exercise)) OR TS=(strength exercise) and Preprint Citation Index (Exclude – Database)  #4=((TS=(female)) OR TS=(woman)) OR TS=(women) and Preprint Citation Index (Exclude – Database)  #5=((((((((((TS=(Single-Blind Method)) OR TS=(Double-Blind Method)) OR TS=(Randomized Controlled Trial)) OR TS=(Clinical Trials)) OR TS=(random allocation)) OR TS=(placebo)) OR TS=(single blind)) OR TS=(double blind)) OR TS=(RCT)) OR TS=(random*)) OR TS=(Randomized Controlled Trials) and Preprint Citation Index (Exclude – Database)  #6=#1 AND #2 AND #3 AND #4AND #5 and Preprint Citation Index (Exclude – Database) |
| Embase | #1 'sarcopenia'/exp OR 'sarcopenia'  #2'sarcopenia':ab,ti OR 'muscular atrophy':ab,ti OR 'muscle weakness':ab,ti OR 'sarcopenic':ab,ti OR 'sarcopeni*':ab,ti  #3 #1 OR #2  #4 'obesity':ab,ti OR 'overweight':ab,ti OR 'obese':ab,ti OR 'obestic':ab,ti OR 'obes*':ab,ti  #5 'resistance training'/exp OR 'resistance training'  #6 'weight lifting':ab,ti OR 'resistance training':ab,ti OR 'resistance exercise':ab,ti OR 'training, resistance':ab,ti OR 'strength training':ab,ti OR 'training, strength':ab,ti OR 'weight-lifting strengthening program':ab,ti OR 'strengthening program, weight-lifting':ab,ti OR 'strengthening programs, weight-lifting':ab,ti OR 'weight lifting strengthening program':ab,ti OR 'weight-lifting strengthening programs':ab,ti OR 'weight-lifting exercise program':ab,ti OR 'exercise program, weight-lifting':ab,ti OR 'exercise programs, weight-lifting':ab,ti OR 'weight lifting exercise program':ab,ti OR 'weight-lifting exercise programs':ab,ti OR 'weight-bearing strengthening program':ab,ti OR 'strengthening program, weight-bearing':ab,ti OR 'strengthening programs, weight-bearing':ab,ti OR 'weight bearing strengthening program':ab,ti OR 'weight-bearing strengthening programs':ab,ti OR 'weight-bearing exercise program':ab,ti OR 'exercise program, weight-bearing':ab,ti OR 'exercise programs, weight-bearing':ab,ti OR 'weight bearing exercise program':ab,ti OR 'weight-bearing exercise programs':ab,ti OR 'lifting, weight':ab,ti OR 'liftings, weight':ab,ti OR 'weight liftings':ab,ti OR 'elastic band':ab,ti OR 'body weight training':ab,ti OR 'strengthening exercise':ab,ti OR 'strength exercise':ab,ti  #7 #5 OR #6  #8 'female'/exp OR 'female'  #9 'women':ab,ti OR 'female':ab,ti OR 'woman':ab,ti  #10 #8 OR #9  #11 'randomized controlled trial'/exp OR 'randomized controlled trial'  #12 'single-blind method':ab,ti OR 'double-blind method':ab,ti OR 'randomized controlled trials':ab,ti OR 'intention to treat analysis':ab,ti OR 'controlled clinical trials':ab,ti OR 'clinical trials':ab,ti OR 'clinical trial':ab,ti OR 'randomized controlled trial':ab,ti OR 'random*':ab,ti OR 'allocation':ab,ti OR 'random allocation':ab,ti OR 'placebo':ab,ti OR 'single blind':ab,ti OR 'double blind':ab,ti OR 'randomized controlled trial*':ab,ti OR 'rct':ab,ti  #13 #11 OR #12  #14 'obesity'/exp OR 'obesity'  #15 #4 OR #14  #16 #3 AND #7 AND #10 AND #13 AND #15 |
| Cochrane Library | #1 MeSH descriptor: [Sarcopenia] explode all trees  #2 (Sarcopenia):ab,ti,kw OR (Muscular Atrophy):ab,ti,kw OR (Muscle Weakness):ab,ti,kw OR (sarcopenic):ab,ti,kw OR (Sarcopeni*):ab,ti,kw  #3 #1 OR #2  #4 (MeSH descriptor: [Obesity] explode all trees  #5 (Obesity):ab,ti,kw OR (Overweight):ab,ti,kw OR (Obese):ab,ti,kw OR (Obestic):ab,ti,kw OR (obes*):ab,ti,kw OR (adiposity):ab,ti,kw  #6 #4 OR #5  #7 MeSH descriptor: [Resistance Training] explode all trees  #8 (Weight Lifting):ab,ti,kw OR (Resistance Training):ab,ti,kw OR (resistance exercise):ab,ti,kw OR (Training, Resistance):ab,ti,kw OR (Strength Training):ab,ti,kw OR (Training, Strength):ab,ti,kw OR (Weight-Lifting Strengthening Program):ab,ti,kw OR (Strengthening Program, Weight-Lifting):ab,ti,kw OR (Strengthening Programs, Weight-Lifting):ab,ti,kw OR (Weight Lifting Strengthening Program):ab,ti,kw OR (Weight-Lifting Strengthening Programs):ab,ti,kw OR (Weight-Lifting Exercise Program):ab,ti,kw OR (Exercise Program, Weight-Lifting):ab,ti,kw OR (Exercise Programs, Weight-Lifting):ab,ti,kw OR (Weight Lifting Exercise Program):ab,ti,kw OR (Weight-Lifting Exercise Programs):ab,ti,kw OR (Weight-Bearing Strengthening Program):ab,ti,kw OR (Strengthening Program, Weight-Bearing):ab,ti,kw OR (Strengthening Programs, Weight-Bearing):ab,ti,kw OR (Weight Bearing Strengthening Program):ab,ti,kw OR (Weight-Bearing Strengthening Programs):ab,ti,kw OR (Weight-Bearing Exercise Program):ab,ti,kw OR (Exercise Program, Weight-Bearing):ab,ti,kw OR (Exercise Programs, Weight-Bearing):ab,ti,kw OR (Weight Bearing Exercise Program):ab,ti,kw OR (Weight-Bearing Exercise Programs):ab,ti,kw OR (Lifting, Weight):ab,ti,kw OR (Liftings, Weight):ab,ti,kw OR (Weight Liftings):ab,ti,kw OR (elastic band):ab,ti,kw OR (body weight training):ab,ti,kw OR (strengthening exercise):ab,ti,kw OR (strength exercise):ab,ti,kw  #9 #7 OR #8  #10MeSH descriptor: [Women] explode all trees  #11 (Women):ab,ti,kw OR (Female):ab,ti,kw OR (woman):ab,ti,kw  #12 #10 OR #11  #13MeSH descriptor: Randomized Controlled Trial] explode all trees  #14(Single-Blind Method):ab,ti,kw OR (Double-Blind Method):ab,ti,kw OR (Randomized Controlled Trials):ab,ti,kw OR (Randomized Controlled Trial):ab,ti,kw OR (Intention to Treat Analysis):ab,ti,kw OR (Controlled Clinical Trials):ab,ti,kw OR (Clinical Trials):ab,ti,kw OR (Clinical Trial):ab,ti,kw OR (randomized controlled trial):ab,ti,kw OR (random*):ab,ti,kw OR (Allocation):ab,ti,kw OR (random allocation):ab,ti,kw OR (Placebo):ab,ti,kw OR (single blind):ab,ti,kw OR (double blind):ab,ti,kw OR (randomized controlled trial*):ab,ti,kw OR (RCT):ab,ti,kw  #15#13 OR #14  #16 #3 AND #6 AND #9 AND #12 AND #15 |
